# miRNA‐regulated transcription associated with mouse strains predisposed to hypnotic effects of ethanol

**DOI:** 10.1002/brb3.989

**Published:** 2018-04-30

**Authors:** B. Vestal, P. Russell, R.A. Radcliffe, L. Bemis, L.M. Saba, K. Kechris

**Affiliations:** ^1^ Center for Genes, Environment and Health National Jewish Health Denver Colorado; ^2^ Department of Biostatistics and Informatics University of Colorado Denver Anschutz Medical Campus Aurora Colorado; ^3^ Department of Pharmaceutical Sciences University of Colorado Denver Anschutz Medical Campus Aurora Colorado; ^4^ Department of Biomedical Sciences University of Minnesota Medical School Duluth Campus Duluth Minnesota

**Keywords:** calcium/calmodulin‐dependent protein kinase II inhibitor 1, ethanol sensitivity, loss of righting reflex, microRNA expression, microRNA target interactions, mmu‐miR‐106b, mouse brain, mRNA expression, recombinant Inbred mouse strains, RNA sequencing

## Abstract

**Introduction:**

Studying innate sensitivity to ethanol can be an important first step toward understanding alcohol use disorders. In brain, we investigated transcripts, with evidence of miRNA modulation related to a predisposition to the hypnotic effect of ethanol, as measured by loss of righting reflex (LORR).

**Methods:**

Expression of miRNAs (12 samples) and expression of mRNAs (353 samples) in brain were independently analyzed for an association with LORR in mice from the LXS recombinant inbred panel gathered across several small studies. These results were then integrated via a meta‐analysis of miRNA–mRNA target pairs identified in miRNA‐target interaction databases.

**Results:**

We found 112 significant miRNA–mRNA pairs where a large majority of miRNAs and mRNAs were highly interconnected. Most pairs indicated a pattern of increased levels of miRNAs and reduced levels of mRNAs being associated with more alcohol‐sensitive strains. For example, *CaMKIIn1* was targeted by multiple miRNAs associated with LORR. CAMK2N1 is an inhibitor of CAMK2, which among other functions, phosphorylates, or binds to GABA_A_ and NMDA receptors.

**Conclusions:**

Our results suggest a novel role of miRNA‐mediated regulation of an inhibitor of CAMK2 and its downstream targets including the GABA_A_ and NMDA receptors, which have been previously implicated to have a role in ethanol‐induced sedation and sensitivity.

## 1 INTRODUCTION

Alcohol use disorders are a major health and societal problem in the United States. More than 18 million Americans meet the diagnostic criteria for alcohol abuse or dependence (Grant et al., 2004). It is estimated that 50%–60% of the variance in alcohol dependence can be attributed to genetic factors (Dick & Foroud, 2002). Some genetic variants relevant to alcohol use disorders may occur in gene coding regions and affect the structure and function of a protein (Johnson et al., 2011). Several candidate genes with these types of variants have been proposed as etiological factors in the development of alcohol use disorders based on association studies of alcohol dependence in humans and other animals, but without a complete understanding of how these gene products may mediate the development of alcohol use disorders (Dick & Bierut, 2006). Other variants may occur in noncoding regions or synonymous codon positions and can affect the regulation of gene or protein expression or the activity of noncoding (nc) RNA. Due to their diverse roles, noncoding polymorphisms are more difficult to characterize, but are often identified in genomewide association studies and have been shown to contribute to disease and clinical phenotypes (Goulart et al., 2015; Musunuru et al., 2010).

One type of ncRNA is a microRNA (miRNA), which is relevant for studying differences in gene expression as it can serve as master regulator of stability and translation of a large number of RNA transcripts. miRNAs typically bind to the 3′ untranslated region (3′ UTR) of target messenger RNA (mRNA) and can facilitate degradation of the transcript or repress subsequent translation (Bartel, 2009). The examination of the role of miRNA has been recognized as an important direction for studying neuropsychiatric disorders such as alcohol dependence (Forero, van der Ven, Callaerts, & Del‐Favero, 2010; Miranda et al., 2010). In particular, the review in Forero et al. (2010) highlights the importance of comprehensive profiling of miRNA expression in genetic models of psychiatric diseases. In rodent neurons, miR‐9 was found to target and promote degradation of specific splice variants of the BK channel (Pietrzykowski et al., 2008). Differences in the relative amount of isoform variants within the BK channel contribute to the channel's tolerance and sensitivity to alcohol (Pietrzykowski et al., 2008). A recent study by Mamdani et al. used coexpression networks consisting of both miRNAs and mRNAs to identify modules correlated with alcohol dependence in the nucleus accumbens of postmortem brain from human subjects with or without alcohol dependence (Mamdani et al., 2015). They found neuronal specific modules enriched for genes involved in oxidative phosphorylation, mitochondrial dysfunction, and MAPK signaling.

Because of the genetic heterogeneity, ethical concerns, and uncontrolled environmental effects in human studies, animal models are an invaluable alternative for studying the genetics of alcohol dependence, including the functional role of miRNAs (Tabakoff & Hoffman, 2000). For example, Nunez et al. found significant conservation in the miRNAs that were differentially expressed in response to ethanol in the frontal cortex of mice as compared to the miRNAs found to be differentially expressed in the prefrontal cortex of postmortem brains from human alcoholics when compared to nonalcoholics (Nunez et al., 2013). Furthermore, animal models are amenable to the study of subtraits, called endophenotypes, which may be critical components to the full expression of a disorder. Measuring endophenotypes that may be functionally related to alcohol dependence on a genetically profiled panel of (typically mammalian) animals has been important for discovering candidate risk factors and genes (Tabakoff & Hoffman, 2000). One well‐studied endophenotype is sensitivity to low doses of alcohol, as first noted by Schuckit (Schuckit, 1980), who observed that individuals who were “family history positive” for alcohol use disorders were reliably less sensitive to the effect of an acute alcohol challenge, compared to those who were “family history negative”, a phenomenon that Schuckit termed “low level of response.” Level of response subsequently has been shown to be heritable (Heath et al., 1999) and to be a reliable predictor of future drinking problems (Schuckit, 1994). Given this finding, studying the molecular factors that influence endophenotypes of initial sensitivity to the high‐dose hypnotic effect of alcohol could be an important step toward understanding the factors that influence the level of alcohol consumption, potentially leading to alcohol use disorders.

The main focus of the work presented here is to identify miRNA–mRNA pairs that are associated with initial sensitivity to the hypnotic effect of ethanol as measured by loss of righting reflex (LORR; (Haughey et al., 2005)) in a well‐characterized mouse recombinant inbred panel. This was achieved by first analyzing mouse miRNA and mRNA expressions aggregated from several small studies independently for an association with LORR, and then combining the results via a meta‐analysis of all miRNA–mRNA pairs that had either a predicted or validated interaction. From this analysis, a final set of candidate pairs with a strong relationship with LORR was investigated in more detail, including experimental validation for a selected interaction.

## 2 MATERIALS AND METHODS

### 2.1 LXS panel

The original long‐sleep (LS) and short‐sleep (SS) lines of mice were selectively bred for a long or short duration of LORR due to ethanol (i.e., sleep time) from a heterogeneous stock that was derived from eight different inbred mouse strains (Markel, Defries, & Johnson, 1995; Williams et al., 2004). The inbred LS (ILS) and SS (ISS) were subsequently generated from the selected lines and were used as founders for creating the LXS (ILSXISS) RI panel (Williams et al., 2004).

**Figure 1:**
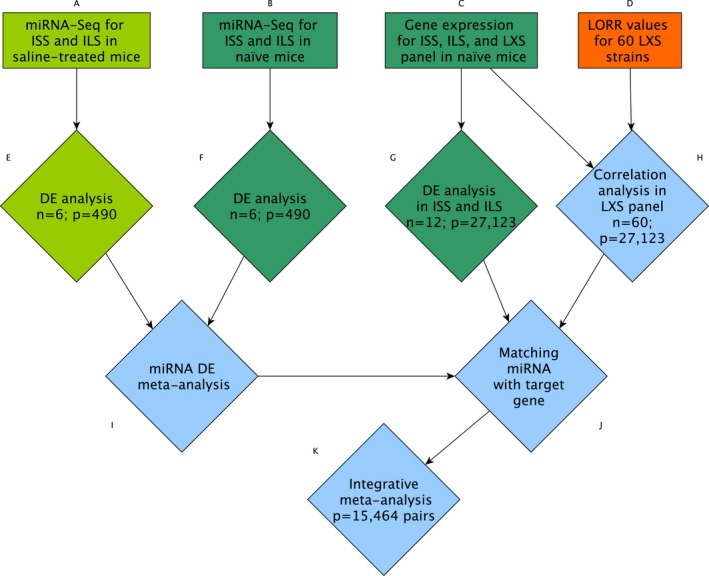
Visualization of the analysis pipeline resulting in the integrative meta‐analysis associating miRNA–mRNA pairs with loss of righting reflex (LORR). Rectangles represent data sources, and diamonds are analysis steps. Light green denotes data and analyses specific to the saline‐treated mice, dark green for the naïve mice, and orange for previously published information. Each blue node is a step where information is integrated from multiple sources. The numbers in the analysis nodes represent the number of samples analyzed (*n*) at that step, and the number of features interrogated (*p*). DE, differential expression

Our work includes data from animals in three separate data sets derived from smaller studies that we looked to integrate via a meta‐analysis. All animal procedures have been established to ensure the absolute highest level of humane care and use of the animals and have followed the National Institutes of Health (NIH) Guide for the Care and Use of Laboratory Animals. All procedures were approved by the University of Colorado Anschutz Medical Campus Institutional Animal Care and Use Committee (IACUC). The first set of mice included the two parental strains of the LXS panel, the ILS and ISS (Figure 1a). In this experiment, male mice (group housed) approximately 9 weeks of age received a saline treatment (Bennett et al., 2015). Mice were administered normal saline (0.01 ml/g) and sacrificed 8 hr later by CO_2_ inhalation followed by decapitation during the light phase. The brain was removed, further dissected into cerebellum and whole brain (minus the olfactory bulbs), and stored in RNALater (Thermo Fisher Scientific, Wilmington, DE, USA) at −20°C until RNA extraction. A total of six mice comprise the saline data set with three for each strain.

In the second data set (Figure 1b,c), RNA from brains of 60 strains of the LXS panel and the two parental strains was analyzed. We will refer to this set of mice as the naïve set. Male mice (group housed) were rapidly sacrificed using CO_2_ gas at approximately 10 weeks of age during the light phase, and brains were removed, divided sagittally, and placed in RNALater (Thermo Fisher Scientific) for RNA extraction and quantitation. In the naïve data set, whole brain miRNA expression was available for the parental strains (*n* = 3 replicates per strain, total of six samples) while whole brain mRNA expression was available for both parental strains and the 60 LXS strains (*n* = 4–6 replicates per strain, total of 353 samples).

For the third data set (Figure 1d), published LORR phenotypic data from the LXS panel were used (Haughey et al., 2005). Briefly, mice were administered an intraperitoneal dose of ethanol and placed on their backs in a V‐shaped tray. The time at which the mice could not right themselves (correct their orientation) was recorded as the initial loss of righting reflex. The time at which the mice could right themselves three times within 1 min was recorded as the time of regaining the righting reflex. LORR was calculated as the difference between the times of regain and initial loss of the righting reflex in minutes. While this data were collected from mice that were unexposed to ethanol prior to the LORR experiment (similar to the naïve mice described above), the LORR values are highly correlated with values collected from saline‐treated mice (Spearman's rank correlation = 0.83; exact *p* < 1e‐6) which suggest that the LORR phenotype is not affected by the saline injection (Bennett et al., 2015).

### 2.2 Sample and data collection

#### 2.2.1 miRNA extraction, sequencing, and quantitation

In the saline‐treated mice, total RNA was collected from whole brain (minus cerebellum and olfactory bulbs), and miRNA libraries were prepared using the Illumina TruSeq Small RNA Sample Prep kit (Illumina, San Diego, CA, USA). Fragments between 20 and 35 base pairs were size selected, and libraries were barcoded and sequenced on a single lane of an Illumina HiSeq 2000 (Illumina). For the naïve data set, total RNA was collected from whole brain (right hemisphere) using the RNeasy Plus Universal Midi, Mini, and MinElute kits for RNA sequencing (Qiagen, Valencia, CA, USA). Again, libraries were prepared using the Illumina TruSeq Small RNA Sample Prep kit (Illumina), and fragments between 20 and 35 base pairs were size selected. Libraries were sequenced on the Illumina HiSeq 2500 platform (Illumina) in three lanes in one batch. Alignment was performed with Bowtie (version 1.1.1; RRID: http://scicrunch.org/resolver/SCR_005476; (Langmead, Trapnell, Pop, & Salzberg, 2009)), and quantitation was performed with RSEM (version 1.2.19; RRID: http://scicrunch.org/resolver/SCR_013027; (Li & Dewey, 2011)) to estimate counts for a miRNA transcriptome (Appendix S1).

#### 2.2.2 mRNA extraction and quantitation

For measures of brain mRNA expression levels (Figure 1c), the public data set on 60 LXS strains and the two parental strains was downloaded from the PhenoGen website (http://phenogen.ucdenver.edu; RRID: http://scicrunch.org/resolver/SCR_001613; (Vanderlinden, Saba, Bennett, Hoffman, & Tabakoff, 2015; Hoffman et al., 2011)). These data were generated using total RNA extracted from whole brain of the same naïve mice as above (using the left hemisphere instead of the right) and using the same extraction procedure as described earlier. Data processing is described in the Appendix S1.

#### 2.2.3 LXS marker set

The original LXS genetic marker set consisting of 303,988 single nucleotide polymorphisms (SNPs) with unique dbSNP identifiers was generated by The Jackson Laboratory using the Affymetrix Mouse Diversity Genotyping Array (Yang et al., 2011) (Appendix S1).

### 2.3 Statistical analysis

The individual steps of the analysis pipeline are illustrated in Figure 1 and detailed in the following subsections. In short, differential expression analyses between the parental strains ISS and ILS were conducted in both of the miRNA data sets (Figure 1a,b) as well as the mRNA data set (Figure 1c). Additionally, a correlation analysis between LORR values and mRNA expression was performed (Figure 1d) in the other LXS strains to focus attention on miRNA–mRNA target pairs associated with LORR as the ISS and ILS may also differ on other phenotypes. Evidence for an association between the curated list of candidate miRNA–mRNA target pairs and LORR was assessed using a meta‐analysis (Figure 1k) that integrated the raw *p*‐values from the individual analyses.

All analyses were performed using R version 3.1.2 (Team, 2000). Although miRNAs target mRNAs, not genes, the analyses are all performed at the gene level because the data resources described below are summarized (mRNA expression) or only available (miRNA‐target database) at the gene level. Adjustments for multiple comparisons were performed using a false discovery rate (FDR) when applicable (Benjamini & Hochberg, 1995).

#### 2.3.1 *miRNA:* differential expression

The two miRNA data sets were analyzed independently because of total confounding between the exposure to saline and experiment (Figure 1e,f). miRNA features were filtered to only include the miRNAs that had at least three samples with a count per million value greater than 1 based on the RSEM quantitation (Rau, Gallopin, Celeux, & Jaffrézic, 2013). A miRNA had to pass this filter in both data sets independently. For the miRNAs passing this filter, separately for the saline‐treated and naïve data sets, a test for differences between strains (ILS vs. ISS) was performed with a quasi‐likelihood ratio test (QuasiSeq R package V1.0‐8; RRID: http://scicrunch.org/resolver/SCR_001715) comparing the model including a strain effect to an intercept‐only model (Lund, Nettleton, Mccarthy, & Smyth, 2012) (Appendix S1). Unadjusted *p*‐values were retained for use in subsequent analyses and were also examined during intermediate analyses to assess the evidence of differential expression between strains from each set. Finally, the unadjusted *p*‐values from tests for differential expression were combined via a meta‐analysis using Stouffer's *Z*‐score method (Rosenthal, 1978) for all miRNAs that passed the counts per million filters in both data sets (Figure 1I).

#### 2.3.2 mRNA: differential expression and LORR correlation

Two separate analyses were performed to investigate the relationship between mRNA expression and LORR. First, differential expression between the parental strains was analyzed using a linear model framework with the limma package (V3.28.17; RRID: http://scicrunch.org/resolver/SCR_010943) in R (Figure 1g) (Smyth, 2005). Second, a Spearman's rank correlation between mRNA expression (using strain means) and LORR was computed for each gene in the LXS panel mice, without the parental strains (Figure 1h).

#### 2.3.3 miRNAs and mRNAs: integrative analysis

The set of all filtered miRNAs was examined with the multiMiR R package V2.1.1 (Ru et al., 2014) to find both validated and predicted targets, reported at the gene level, for each miRNA. All validated pairs that appeared in at least one database and the predicted target interactions that appeared in at least five databases of eight were retained for further analysis (Figure 1j). For each of the miRNA–mRNA target pairs, the unadjusted *p*‐values from the three different analyses (miRNA differential expression, mRNA differential expression, and mRNA–LORR correlation) were combined using the Stouffer's Z‐score method (Rosenthal, 1978) to obtain an integrative meta‐analysis *p*‐value for that pair (Figure 1k), which were then adjusted for multiple comparisons using a FDR threshold of 0.10 (Appendix S1).

#### 2.3.4 Further characterization of miRNA–mRNA pairs

All of the unique mRNAs and miRNAs in the significant miRNA–mRNA pairs from the integrative meta‐analysis were tested for statistical enrichment of pathways (Appendix S1). Additionally, for each pair, the location of the miRNAs in the genome was compared with both the associated mRNA's expression (e)quantitative trait locus (if a significant one was found) and the LORR QTLs (Appendix S1). Additional summaries were tabulated for the significant pairs (Appendix S1).

#### 2.3.5 Validation of selected predicted targets

A miRNA–gene pair with one of the strongest integrated meta‐analysis *p*‐values was selected for experimental validation of the predicted relationship. A luciferase assay was performed where the predicted mmu‐miR‐106b‐5p binding site in *CaMKIIn1* (calcium/calmodulin‐dependent protein kinase II inhibitor 1) was placed in the plasmid pSI‐CHECK2 and expressed in HEK293T cells to confirm binding of miR‐106b‐5p to *CaMKIIn1* (Appendix S1).

## 3 RESULTS

### 3.1 Differential expression of miRNA

Approximately 9 to 12 million (M) RNA‐Seq reads per sample after clipping and quality filtering were retained from the small RNA fraction in the saline‐treated mice. Of these reads, the majority aligned to miRNAs (57%; Table S1). The same process for the naïve mice resulted in about 15M–30M reads per sample with the majority aligning to miRNAs (62%; Table S1). Only miRNA features were quantified using RSEM and used for statistical analyses. A total of 1,915 miRNAs were interrogated with 490 of these passing the counts per million filter in both of the data sets. The median miRNA library sizes were 2.6M and 6.4M counts for saline‐treated and naïve samples, respectively, using estimated counts from RSEM for only miRNAs (Table S1).

There were 45 and 40 miRNAs with a *p*‐value less than .05 for differential expression between strains (Figure 1e,f) in the saline‐treated and naïve data sets, respectively. Seven miRNAs were differentially expressed in both data sets, and for five of the seven, the direction of the effect was consistent (Table 1). Although the magnitude of the overlap between the two data sets was not large due to our strict criteria (*p*‐value < .05 for both sets) and limited sample size in each of the studies, there was overall concordance between the two studies with more relaxed thresholds (Figures S1 and S2). For example, by classifying miRNAs as either significantly upregulated in ILS (*p*‐value < .20 and DE in the correct direction) or not significantly upregulated in ILS, there was a significant association between the two studies in their classification of the miRNAs (Fisher's exact test *p*‐value = 1.8e‐4). Performing this same process but instead classifying miRNAs as either upregulated in ISS or not gave similar results (Fisher's exact test *p*‐value = .02). Moreover, when comparing all pairwise Spearman correlations between the 12 samples (six saline‐treated and six naïve), the smallest value was 0.91 while the bulk of the correlations was around 0.95–0.98. This also suggests that the overall expression profiles in terms of miRNA in whole brain are relatively stable across the saline‐treated and naïve mice.

**Table 1:** miRNAs differentially expressed between inbred short‐sleep (ISS) and inbred long‐sleep (ILS) in both data sets and in the same direction. Strain effects are reported as expression of ISS as a percentage of ILS

miRNA	Saline strain effect *p*‐value	Naïve strain effect *p*‐value	Saline strain effect	Naïve strain effect	miRNA meta‐analysis *p*‐value	miRNA meta‐analysis FDR
mmu‐miR‐5121	.0153	.0016	28%	29%	.0001	0.0383
mmu‐miR‐219a‐2‐3p	.0147	.0115	80%	59%	.0004	0.0538
mmu‐miR‐155‐5p	.0044	.0342	236%	195%	.0004	0.0538
mmu‐miR‐219b‐5p	.0243	.0160	60%	49%	.0010	0.0696
mmu‐miR‐668‐3p	.0475	.0356	82%	65%	.0039	0.1424

When combining across the two data sets in the miRNA‐specific meta‐analysis, we found 61 miRNAs with a significant meta‐analysis *p*‐value < .05. Of the 61 miRNAs, 33 (54%) were upregulated in ISS (based on an average effect between saline‐treated and naïve analyses), and 28 (46%) were upregulated in ILS. See Table S2 for the full list of miRNA‐specific results.

### 3.2 mRNAs associated with LORR

After normalization, 27,123 mRNAs were analyzed for differential expression between the parental strains and for correlation with LORR in the LXS panel mice. A total of 979 mRNAs had an FDR less than 0.05 for differential expression between ISS and ILS mice (Figure 1g). The mRNA with the smallest unadjusted *p*‐value was *Fggy (*FGGY carbohydrate kinase domain containing; expression in ISS 139% higher than ILS, *t*_11_^ ^= 17.04, *p* < 1e‐9; FDR = 5.17e‐6), which phosphorylates carbohydrates (Skarnes et al., 2011). A total of 785 mRNAs had a *p*‐value less than .05 (though none had an FDR < 0.05) in terms of the correlation between expression and LORR (Figure 1h). The strongest observed correlation was with *Lonrf3* (LON peptidase N‐terminal domain and ring finger 3; *r* = −.54, exact *p *= 8.3e‐6; FDR = 0.22), known to be involved in protein–protein and protein–DNA interactions. Thirty‐three mRNAs overlap between the two lists (i.e., both correlated with LORR and differentially expressed in the parental strains) and are detailed in Table 2 with 18 (55%) and 15 (45%) showing a positive or negative correlation with LORR, respectively. See Table S3 for the full set of mRNA‐specific results.

**Table 2:** Summary of mRNA associated with LORR in both data sets (FDR < 0.05 in the differential expression analysis and an unadjusted *p*‐value < .05 in the correlation analysis with LORR)

Associated gene name	Strain effect	Strain effect FDR	Correlation between expression and LORR	Correlation *p*‐value
Hjurp	47%	<0.0001	0.26	.0429
Camk2n1	57%	<0.0001	0.26	.0475
Myo1d	44%	<0.0001	0.29	.0264
Gm6969	328%	<0.0001	−0.31	.0186
Morn2	38%	<0.0001	0.28	.0293
2610044O15Rik8	36%	0.0001	0.26	.0434
Tceanc2	157%	0.0006	0.30	.0217
Pqlc3	64%	0.0017	0.30	.0225
Gns	126%	0.0023	−0.27	.0417
Slc25a24	135%	0.0029	−0.26	.0428
Itgb1 bp1	69%	0.0045	0.27	.0378
Zbbx	129%	0.0078	−0.28	.0334
Dzip3	131%	0.0092	−0.28	.0329
Pemt	65%	0.0104	0.26	.0449
Zfp386	127%	0.0139	−0.28	.0298
Plce1	140%	0.0139	−0.26	.0491
Spata6	134%	0.0179	0.26	.0462
Alyref2	137%	0.0186	−0.27	.0351
1110015O18Rik	179%	0.0228	−0.29	.0246
Cmtm6	130%	0.0252	−0.40	.0018
B3gat2	82%	0.0256	0.27	.0357
Greb1	83%	0.0256	0.27	.0424
Gm14637	61%	0.0285	0.26	.0431
Abi3 bp	63%	0.0287	0.27	.0422
Mpp4	147%	0.0375	−0.29	.0272
Smpdl3b	78%	0.0411	−0.26	.0462
Gm16556	72%	0.0422	−0.29	.0270
Dthd1	145%	0.0443	−0.30	.0227
Rps8‐ps1	250%	0.0471	0.32	.0134
9130020K20Rik	74%	0.0474	0.27	.0361
Ephx4	80%	0.0474	0.28	.0328
Mir200a	70%	0.0477	−0.33	.0104
Glul	69%	0.0494	0.35	.0063

FDR, false discovery rate.

The strain effect is reported as expression of ISS as a percentage of ILS.

### 3.3 Results of integrative analysis of miRNAs and target mRNAs

**Figure 2:**
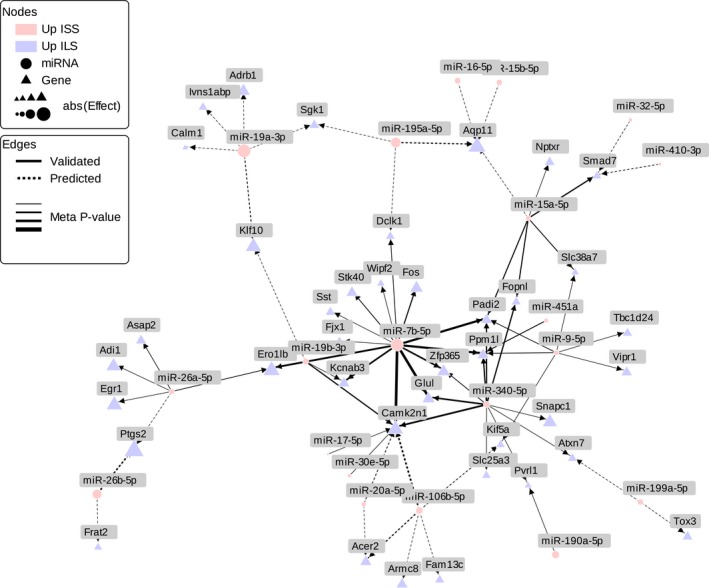
Network diagram of connections between miRNAs and mRNAs for the most interconnected region from the 112 miRNA–mRNA pairs associated with LORR in the integrative meta‐analysis. Circles represent miRNAs while triangles are mRNA denoted by their gene name. The line type denotes the type of mRNA–miRNA interaction (validated are solid, and predicted are dashed), and the line weight represents the strength of the integrative meta‐analysis *p*‐value for that pair (thicker lines have smaller *p*‐values). The color of the features represents the direction of the effect from the individual analyses (i.e., differential expression) with red denoting overexpression in ISS and blue overexpression in ILS. The size of the nodes is proportional to the magnitude (abs, absolute value) of the log fold change in the individual differential expression analysis between the parental strains for a given feature (averaged across the two data sets for miRNAs)

**Figure 3:**
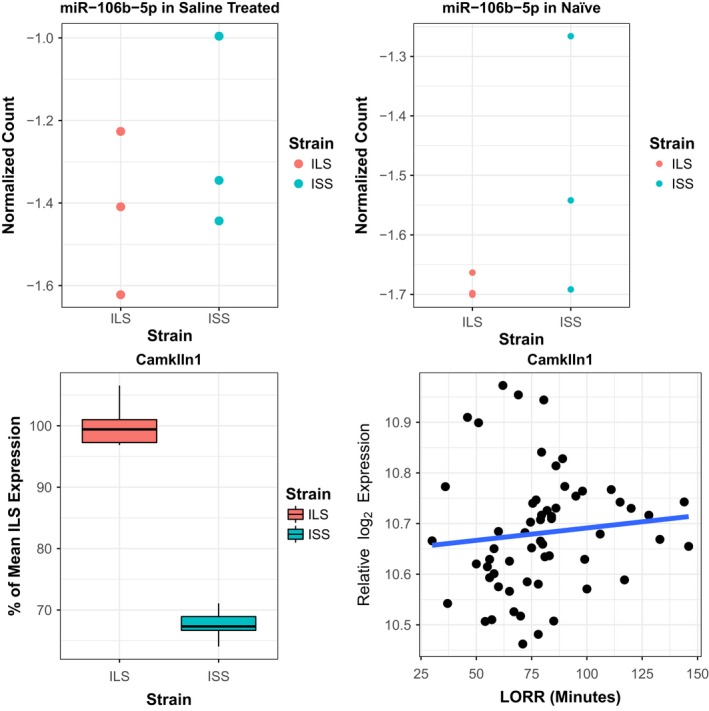
Visual summaries of the four sources of information used to generate the integrative meta‐analysis *p*‐value for mmu‐miR‐106b‐5p and *CaMKIIn1*. The top row contains dot plots for miRNA expression in the parental strains for the saline‐treated mice (left) and the naïve mice (right). The miRNA expression is displayed in terms of normalized counts that represent the scale on which the generalized linear models used for analysis operated. These normalized counts were obtained by dividing the observed counts from each sample by the 75th percentile of counts across all miRNAs in that sample, and these values were then natural log transformed. In the bottom left corner are box plots with mRNA expression in the parental strains from the Affymetrix exon array. In the bottom right corner is a plot of mRNA expression versus loss of righting reflex in the LXS panel mice with the fitted regression line

**Figure 4:**
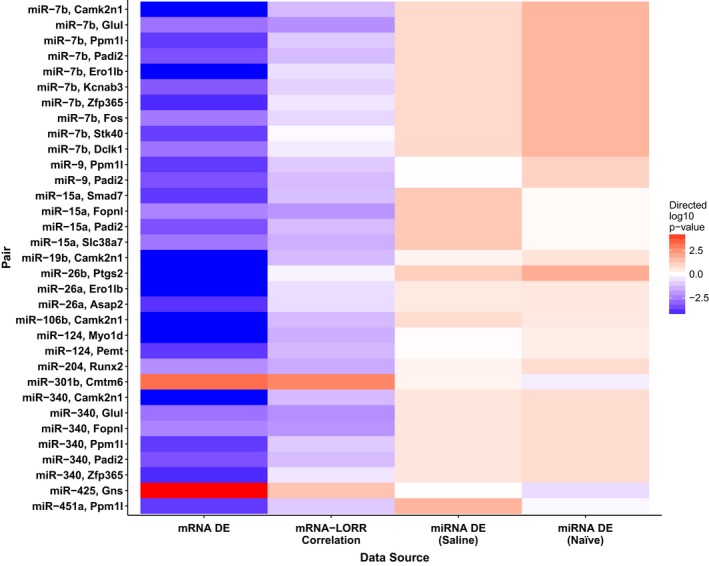
Strength of evidence from the four data sources that are included in the integrative meta‐analysis *p*‐value for the top 33 pairs with an FDR <0.05 (sorted by miRNA identifier). The intensity of the color in a given cell is proportional to the negative log base 10 transformation of the unadjusted *p*‐value for the respective analysis (i.e., darker cells have stronger evidence). The direction of the effect is represented by the color: blue denotes overexpression in ILS (or positive correlation with loss of righting reflex) while red represents overexpression in ISS. DE, differential expression

The multiMiR query for miRNA–mRNA interactions returned 8,429 validated interactions and 7,035 predicted interactions. In total, 268 (55%) of the 490 miRNAs we evaluated had predicted or validated targets returned from the multiMiR query. The integrative meta‐analysis identified a total of 112 miRNA–mRNA pairs with an FDR < 0.10 (Figure 1k), of which 51 (46%) were from validated targets and the remaining 61 (54%) were from predicted interactions (visualized with an interactive network diagram at https://goo.gl/b2ClPe with a highly connected region of 70 pairs of the 112 shown in Figure 2). Of the 112 pairs, there is a strong bias toward the pattern of an upregulated miRNA in ISS, upregulation of the target mRNA in ILS, and a positive correlation between mRNA expression and LORR; this occurred in 89 (79%) of the 112 identified pairs. There were 48 unique miRNAs involved in these 112 pairs where the maximum number of targets for a single miRNA was 13 (mmu‐miR‐7b‐5p). Additionally, there were 75 unique mRNAs represented in the mRNA list with the maximum number of miRNAs targeting a single mRNA being 7 (*CaMKIIn1* – calcium/calmodulin‐dependent protein kinase II inhibitor 1). Figure 3 is an example of the four sources of information being integrated for the pair with the smallest integrative meta‐analysis *p*‐value, mmu‐miR‐106b‐5p, and *CaMKIIn1*. Figure 4 includes the *p*‐values from the 4 individual analyses for the 33 (29%) pairs that had a meta‐analysis FDR < 0.05 of the 112 that had a meta‐analysis FDR < 0.10.

### 3.4 Further characterization of miRNA–mRNA pairs

A summary of a selection of the additional characteristics of the top 33 pairs (meta‐analysis FDR < 0.05) can be found in Table 3 (for the remaining significant pairs see Table S4). We identified two new LORR QTLs, one on chromosome 3 and the other on chromosome 14 (see Tables S4 and S7 for more details). When comparing the locations of the miRNAs in the significant miRNA–mRNA pairs to these two LORR QTLs, there were three miRNAs whose physical location overlapped with the one on chromosome 14 (mmu‐miR‐15a‐5p, mmu‐miR‐16‐5p, and mmu‐miR‐124‐3p); none overlapped with the QTL on chromosome 3. Five of the miRNAs within the significant pairs overlapped with one of the previously discovered LORR QTLs (mmu‐miR‐22‐3p, mmu‐let‐7c‐5p, mmu‐miR‐21a‐5p, mmu‐miR‐128‐3p, mmu‐miR‐451a, and mmu‐miR‐30e‐5p). We did not find any pairs with the physical location of the DNA coding region for the miRNA overlapping the associated mRNA's eQTL.

**Table 3:** Summary of miRNA–mRNA pairs associated with loss of righting reflex (LORR) (integrative meta‐analysis FDR < 0.05)

miRNA	Target symbol	Integrative meta‐analysis FDR	Interaction type	miRNA in sleep time QTL?	Panther pathways	GO biological processes	GO molecular function
mmu‐miR‐7b‐5p	Camk2n1	0.003	Validated	No	CCKR signaling map (1/3)	Epidermal cell differentiation (1/5); regulation of cellular process (13/49); transmembrane receptor protein serine:threonine kinase signaling pathway (2/6)	RNA polymerase II core promoter sequence‐specific DNA binding (1/3); core promoter binding (2/5); core promoter sequence‐specific DNA binding (1/4)
	Glul	0.003	Validated				
	Ppm1 l	0.0047	Validated				
	Padi2	0.0047	Validated				
	Ero1 lb	0.0084	Validated				
	Kcnab3	0.0098	Validated				
	Zfp365	0.0098	Validated				
	Fos	0.0221	Validated				
	Stk40	0.0362	Validated				
	Dclk1	0.0385	Validated				
mmu‐miR‐9‐5p	Ppm1 l	0.0385	Validated	No	Alzheimer disease–presenilin pathway (1/3)	T‐cell activation (1/4); regulation of cellular process (6/49); regulation of synapse structure or activity (2/7); transmembrane receptor protein serine:threonine kinase signaling pathway (2/6)	RNA polymerase II core promoter sequence‐specific DNA binding (1/3); core promoter binding (1/5); core promoter sequence‐specific DNA binding (1/4)
	Padi2	0.0385	Validated				
mmu‐miR‐15a‐5p	Smad7	0.0174	Validated	Yes		Epidermal cell differentiation (1/5); regulation of cellular process (8/49); transmembrane receptor protein serine:threonine kinase signaling pathway (1/6)	
	Fopnl	0.0229	Validated				
	Padi2	0.0229	Validated				
	Slc38a7	0.0309	Validated				

FDR, false discovery rate.

In the columns for panther pathways and gene ontology (GO) terms, the pathway or ontology term is related to at least one gene targeted by the given miRNA.

The numbers in parentheses represent the number of genes that the specific miRNA in that pair targets followed by the total number of genes in that process found to be associated with LORR.

Starting with the list of 48 unique miRNAs from the 112 pairs in the meta‐analysis (FDR < 0.10), we performed pathway enrichment on database targets of these miRNAs and examined miRNA hubs that were highly connected to at least five mRNAs (mmu‐miR‐7b‐5p, mmu‐miR‐340‐5p, mmu‐miR‐19a‐5p, mmu‐miR‐26a‐5p, mmu‐miR‐106b‐5p mmu‐miR‐15a‐5p, and mmu‐miR‐9‐5p). These miRNAs are enriched for target genes in KEGG pathways such as “gap junction (map04540),” “axon guidance (map04360),” “prion diseases (map05020),” “GABAergic synapse (map04727),” “glutamatergic synapse (map04724),” “neurotrophin signaling pathway (map04722),” and “cerebellar long‐term depression (map04730),” which are related to neurological development and disorders. In addition, for mmu‐miR‐9‐5p, several addiction‐related pathways also appeared (cocaine—map05030 and amphetamine—map05031). See Table S5 for miRPath results.

Next, we performed a pathway and gene ontology enrichment analyses on our mRNA lists (Panther; RRID: http://scicrunch.org/resolver/SCR_004869) and found that the panther pathway gene set most frequently targeted by at least one miRNA in the 112 meta‐analysis pairs is the “Alzheimer disease presenilin pathway (P00004)” (enrichment FDR < 0.10). Indeed, six (12.5%) of the 48 miRNAs have a predicted or validated target gene in that pathway. In terms of GO biological processes, we see many categories related to development and synapse structure including “neuron development” (GO:0048666) and “regulation of synapse structure” (GO:0050803). One potentially interesting miRNA is mmu‐miR‐7b‐5p which targets genes in several of these processes (among others). For GO molecular functions, we found that genes in the category “core promoter sequence‐specific DNA binding” (GO:0001046) were targeted by 13 miRNAs (including miR‐7b) in addition to several other promoter binding categories. We also found “calcium‐dependent protein binding” (GO:0048306), which is relevant for neuronal transmission. See Table S6 for the full Panther results.

### 3.5 Validation of selected predicted targets

**Figure 5:**
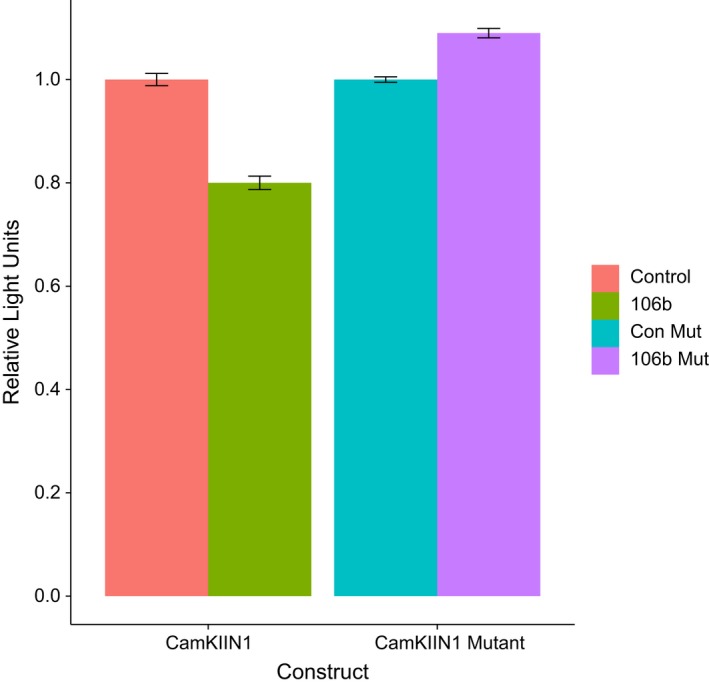
Results of luciferase reporter assay for mmu‐miR‐106b‐5p and *CaMKIIn1*. The first construct (wt) contains the wild‐type mmu‐miR‐106b‐5p predicted binding site, and the second construct contains site‐directed mutations of the predicted binding site. HEK293T cells were transfected with either wild type or mutant plasmids and also with either control (con) or mmu‐miR‐106b‐5p mimics. Bars indicate the standard error. The forward wild‐type *CaMKIIn1* target site is GCACTTT, and the mutant target site is GCAtTcT, where lower case indicates the mutations

mmu‐miR‐106b‐5p was of particular interest because it appeared to regulate multiple gene targets across many of the identified biological processes. The large number of miRNAs predicted to target *CaMKIIn1* suggests that it could be regulated by a network of miRNAs with additive effect. We used a luciferase reporter assay in HEK293T cells to evaluate the binding of mmu‐miR‐106b‐5p to the 3′UTR of *CaMKIIn1* an inhibitor of CAMKII. The assay confirmed that there was significant reduction in luciferase expression when treated with a mimic of mmu‐miR‐106b‐5p compared with the negative control mimic (*Z* = 2.75, *p*‐value < .05; Figure 5). We also mutated the binding site to show that the regulation of the mutated gene by a mimic of mmu‐miR‐106b‐5p is less effective (Figure 5).

## 4 DISCUSSION

Our study diverges from previous studies of miRNAs in alcohol research due to three study design features. First, compared to previously used miRNA microarrays, we employ high‐throughput sequencing experiments for profiling, which provide more comprehensive and sensitive profiling of miRNA expression (Git et al., 2010). Second, much of the published research in this area focuses on differences in miRNA and mRNA expression in the brain after acute and/or chronic ethanol exposure (e.g. Nunez & Mayfield, 2012; Nunez et al., 2013; Lewohl et al., 2011). Instead, we are interested in characterizing baseline genomic features associated with the predisposition to initial sensitivity to alcohol‐induced hypnosis prior to any ethanol‐induced modifications. Third, we combined miRNA and mRNA results that showed differences in brain expression via a meta‐analysis of all miRNA–mRNA pairs that had either a predicted or validated interaction. This analysis resulted in candidate pairs with combined evidence for differences in expression in the ILS and ISS mice (for miRNAs and mRNAs) and a relationship with LORR in the LXS recombinant inbred strains (mRNAs).

Eighty‐nine of the 112 significant miRNA–mRNA pairs that were identified in the integrated meta‐analysis had a pattern of upregulation of the miRNA in ISS, downregulation of the target mRNA in ISS (i.e., upregulated in the ILS), and a positive correlation between mRNA expression and LORR (i.e., higher mRNA expression is associated with higher LORR). These pairs also tended to be highly connected to each other (see network graphs in Figure 2 and interactive graph at https://goo.gl/b2ClPe). In contrast, the few miRNA–mRNA pairs with the opposite direction tended to be isolated, in that the corresponding miRNAs had few targets or the mRNAs were not targeted by other miRNAs. This suggests that miRNAs may be more highly expressed and functional in strains predisposed to be less sensitive to ethanol and that there may be a shared or coordinated activity by multiple miRNAs.

The network generated from our meta‐analysis (Figure 2) also identified several miRNA hubs that were highly connected to at least five mRNAs (mmu‐miR‐7b‐5p, mmu‐miR‐340‐5p, mmu‐miR‐19a‐5p, mmu‐miR‐26a‐5p, mmu‐miR‐106b‐5p, mmu‐miR‐15a‐5p, and mmu‐miR‐9‐5p). Most of these miRNAs are enriched for target mRNAs related to neurological development and synapse function. Of particular interest, mmu‐miR‐15a‐5p was found to overlap with the LORR QTL discovered on chromosome 14 suggesting that the association between genetic variants and LORR may be mediated by SNPs affecting miRNA expression. At the gene level, the most enriched pathway was the “Alzheimer disease presenilin pathway.” While previous work has indicated that there may be increased sensitivity to the sedative effects of ethanol with advanced age (Little, Kuhn, Wilson, & Swartzwelder, 1996), it is unclear why an aging‐related pathway should be informative about innate sensitivity to the sedative effects of ethanol as measured by LORR. One potential explanation for this finding is that the pathway might not have anything to do with the alcohol response, but instead there is a correlated trait in these mice for a susceptibility to Alzheimer's that has a similar genetic underpinning as the trait the selected lines were bred for.

Through the network reconstruction, we also identified the most highly connected mRNA *CaMKIIn1*, which was targeted (either predicted or validated) by seven miRNAs (mmu‐miR‐7b‐5p, mmu‐miR‐340‐5p, mmu‐miR‐19b‐3p, mmu‐miR‐106b‐5p, mmu‐miR‐20a‐5p, mmu‐miR‐30e‐5p, and mmu‐miR‐17‐5p). The protein product CAMKIIn1 interacts with CAMKIIα and CAMKIIβ and inhibits CAMKII, which is involved with brain plasticity (Shonesy, Jalan‐Sakrikar, Cavener, & Colbran, 2014), plays a key role in learning and memory, and interacts with NMDA receptors (Lisman, Yasuda, & Raghavachari, 2012; Shipton & Paulsen, 2014). In a human study, CAMKII has been linked to the establishment of drinking behavior through autophosphorylation of CaMKIIα, and SNPs in CaMKIIα are associated with alcohol dependence (Easton et al., 2013).

CAMKII directly phosphorylates the α1β3 and α1β3γ2S subunits of the gamma‐aminobutyric acid A receptor (GABA_A_) (Houston, Lee, Hosie, Moss, & Smart, 2007). Two of the miRNAs (in our final list of 48 unique miRNAs) were enriched with gene targets for the “GABAergic synapse” pathway, mmu‐miR‐425‐5p and mmu‐miR‐9‐5p, with the latter being one of the most highly connected miRNA in our results. The phosphorylation of GABA_A_ receptors is important for subunit trafficking and membrane stability (Comenencia‐Ortiz, Moss, & Davies, 2014). GABA_A_ receptors are sensitive to alcohol, enhance the effect of ethanol sedation (Silveri & Spear, 2002), and mediate the behavioral effects of ethanol (Olsen, Hanchar, Meera, & Wallner, 2007).

Our results suggest a role for miRNA modulation of GABA_A_ receptors through the inhibitor CAMKIIn1. In all cases, the miRNAs found to target *CaMKIIn1* in our meta‐analysis are upregulated in the ISS strains compared with the more sensitive ILS strains, while the inhibitor *CaMKIIn1* was relatively downregulated in the less sensitive ISS strains compared with the more sensitive ILS strain. This would suggest that in the more sensitive strains, *CaMKIIn1* is not targeted by miRNAs and inhibits *CAMKII*, affecting phosphorylation and functioning of GABA_A_ receptors, and their sensitivity to ethanol. Further studies are needed to confirm direct effects.

Like GABA_A_ receptors, NMDA receptors may also enhance the effect of ethanol sedation (Silveri & Spear, 2002). In our final list, we found three miRNAs (mmu‐miR‐124‐3p, mmu‐miR‐425‐5p, and mmu‐miR‐9‐5p) with targets enriched in “glutamatergic synapse,” which are defined as synapses whose postsynaptic membrane contains glutamate receptors, such as NMDA (Purves et al., 2008). Two of these miRNAs (mmu‐miR‐425‐5p and mmu‐miR‐9‐5p) were also enriched in the “GABAergic synapse” pathway discussed above. NMDA receptors can activate CAMKII, but activated CAMKII can also bind to NMDA receptor subunits and other synaptic proteins to localize its activity to specific regions (Lucchesi, Mizuno, & Giese, 2011). Our results suggest an alternative mechanism of miRNA‐mediated modulation of the hypnotic effects of ethanol through NMDA receptors, in addition to the GABA_A_ receptors.

We tested for binding of mmu‐miR‐106b‐5p to *CaMKIIn1* and confirmed that the predicted miRNA–gene pair, mmu‐miR‐106b‐5p, and *CaMKIIn1* were able to interact to regulate gene expression. These studies were conducted in the readily transfected cell line, HEK293T, and not in a neuronal cell line. Interestingly, the addition of mmu‐miR‐106b‐5p to HEK293T cells caused a reduction in expression of the luciferase constructs suggesting that when mmu‐miR‐106b‐5p is over expressed by the addition of the mimic, the transfected cells are lost. The mechanism of this loss of expression is not clear, and it is unknown if this loss would be observed in a neuronal cell line; this possibility will be the focus of future studies. It could potentially be consistent with slowing of cell growth or even cell death. In this case, it would suggest that when mmu‐miR‐106b‐5p and its target are coexpressed cell viability is altered. This would be a significant finding if it proves to be true in future studies of additional cell lines of brain tissue origin.

Due to small sample sizes in each miRNA study, we used a meta‐analysis approach to combine results from the individual naïve and saline‐treated studies for miRNA differential expression. The phenotype and mRNA expression measurements across the recombinant inbred panel and the different miRNA expression studies in ILS and ISS were performed at different times. However, the nature of the recombinant inbred panel and inbred strains ensures that the genetic makeup of the strains remains relatively consistent. In particular, LORR values in the LXS panel from naïve animals and saline‐injected animals were published in 2005 and 2015, respectively (Bennett et al., 2015; Haughey et al., 2005). Despite the time range and different control conditions, the correlation between LORR values in the two experiments was high (Spearman's rank correlation 0.84, exact *p*‐value < 1e‐6), which supported the integration of expression data from both miRNA data sets in the meta‐analysis. Moreover, the high correlations in overall miRNA expression between all 12 samples (at least 0.91 correlation between any pair of mice) gave further credence to combining the two sets of mice. As the gene expression values for the recombinant inbred panel were from naïve mice only, we used the LORR value from a similar study (Haughey et al., 2005). Additional considerations regarding concordance between the experiments, and the use of male mice and whole brain, are discussed in the Appendix S1.

Another limitation of our study is the reliance on databases to construct the set of possible miRNA–mRNA pairs to be analyzed. As not all of the databases queried by the multiMiR package are readily maintained and updated, we are potentially biasing our results away from more recently discovered miRNAs. Furthermore, database entries are aggregated across all experiments and are not specific to miRNAs and mRNAs expressed specifically in brain. Even the ones that are listed as validated interactions were likely performed in tissue other than mouse whole brain, and there is no guarantee the miRNA targeting behaves in the same way across tissues. Finally, the method of filtering the set of predicted interactions by requiring a consensus of at least five databases significantly reduced the number of pairs we ultimately examined in the meta‐analysis. This threshold left about 7,000 pairs to investigate versus on the order of 500,000 that showed up in at least one database. This resulted in some miRNAs with strong evidence of differential expression being dropped from consideration in the integrative analysis, but the reduction of predicted pairs was deemed necessary to focus on pairs with strong evidence of binding. Thus, relying on these databases was an important filtering step to focus our investigation, and we can only confirm direct targeting in the pair we experimentally validated.

Finally, the meta‐analysis technique we used also took into account the direction of the effect observed in the individual features, so we only focused on pairs where the miRNA and mRNA expressions were negatively associated. Previous studies have examined positively correlated miRNA–mRNA interactions where the working theory was that the positive relationship was a result of the miRNA expression being increased as a regulatory response (Nunez et al., 2013; Pasquinelli, 2012). We instead focused on predisposition to initial sensitivity to the hypnotic effects of alcohol and thus found it more relevant to examine miRNA–mRNA interactions where the target mRNA is suppressed by miRNA in an ethanol naïve setting.

In conclusion, our results indicate that the activity of several miRNAs expressed in the brain may be mediating genetic differences in initial sensitivity to the hypnotic effects of ethanol. These miRNAs tend to be upregulated in the more sensitive strains and coupled with a downregulation of their target mRNAs. In particular, six of the miRNAs are highly connected to many mRNAs, and these miRNAs also targeted many of the same mRNAs. Our results suggest a novel role of miRNA‐mediated regulation of the GABA_A_ and NMDA receptors, which have been previously implicated to have a role in ethanol sedation and sensitivity. This work demonstrates that the role of miRNA regulation is not limited to acute and chronic ethanol exposure, but may also mediate the predisposition to alcohol responses.

## References

[brb3989-bib-0002] Bartel, D. P. (2009). MicroRNAs: Target recognition and regulatory functions. Cell, 136, 215–233. https://doi.org/10.1016/j.cell.2009.01.002 19167326 10.1016/j.cell.2009.01.002 PMC3794896

[brb3989-bib-0003] Benjamini, Y. , & Hochberg, Y. (1995). Controlling the false discovery rate: A practical and powerful approach to multiple testing. Journal of the Royal Statistical Society Series B (Methodological), 57, 289–300.

[brb3989-bib-0005] Bennett, B. , Larson, C. , Richmond, P. A. , Odell, A. T. , Saba, L. M. , Tabakoff, B. , … Radcliffe, R. A. (2015). Quantitative trait locus mapping of acute functional tolerance in the LXS recombinant inbred strains. Alcoholism: Clinical and Experimental Research, 39, 611–620. https://doi.org/10.1111/acer.12678 10.1111/acer.12678 PMC4384187 25833023

[brb3989-bib-0008] Comenencia‐Ortiz, E. , Moss, S. J. , & Davies, P. A. (2014). Phosphorylation of GABAA receptors influences receptor trafficking and neurosteroid actions. Psychopharmacology (Berl), 231, 3453–3465. https://doi.org/10.1007/s00213-014-3617-z 24847959 10.1007/s00213-014-3617-z PMC4135009

[brb3989-bib-0009] Dick, D. M. , & Bierut, L. J. (2006). The genetics of alcohol dependence. Current Psychiatry Reports, 8, 151–157. https://doi.org/10.1007/s11920-006-0015-1 16539893 10.1007/s11920-006-0015-1

[brb3989-bib-0010] Dick, D. M. , & Foroud, T. (2002). Genetic strategies to detect genes involved in alcoholism and alcohol‐related traits. Alcohol Research & Health, 26, 172. 12875045 PMC6683840

[brb3989-bib-0011] Easton, A. C. , Lucchesi, W. , Lourdusamy, A. , Lenz, B. , Solati, J. , Golub, Y. , … Dawirs, R. R. (2013). αCaMKII autophosphorylation controls the establishment of alcohol drinking behavior. Neuropsychopharmacology, 38, 1636–1647. https://doi.org/10.1038/npp.2013.60 23459588 10.1038/npp.2013.60 PMC3717547

[brb3989-bib-0012] Forero, D. A. , van der Ven, K. , Callaerts, P. , & Del‐Favero, J. (2010). miRNA genes and the brain: Implications for psychiatric disorders. Human Mutation, 31, 1195–1204. https://doi.org/10.1002/humu.21344 20725930 10.1002/humu.21344

[brb3989-bib-0014] Git, A. , Dvinge, H. , Salmon‐Divon, M. , Osborne, M. , Kutter, C. , Hadfield, J. , … Caldas, C. (2010). Systematic comparison of microarray profiling, real‐time PCR, and next‐generation sequencing technologies for measuring differential microRNA expression. RNA (New York, N.Y.), 16, 991–1006. https://doi.org/10.1261/rna.1947110 10.1261/rna.1947110 PMC2856892 20360395

[brb3989-bib-0015] Goulart, L. F. , Bettella, F. , Sønderby, I. E. , Schork, A. J. , Thompson, W. K. , Mattingsdal, M. , … Dale, A. M. (2015). MicroRNAs enrichment in GWAS of complex human phenotypes. BMC Genomics, 16, 1. 25884492 10.1186/s12864-015-1513-5 PMC4437677

[brb3989-bib-0016] Grant, B. F. , Dawson, D. A. , Stinson, F. S. , Chou, S. P. , Dufour, M. C. , & Pickering, R. P. (2004). The 12‐month prevalence and trends in DSM‐IV alcohol abuse and dependence: United States, 1991–1992 and 2001–2002. Drug and Alcohol Dependence, 74, 223–234. https://doi.org/10.1016/j.drugalcdep.2004.02.004 15194200 10.1016/j.drugalcdep.2004.02.004

[brb3989-bib-0017] Haughey, H. M. , Kaiser, A. L. , Johnson, T. E. , Bennett, B. , Sikela, J. M. , & Zahniser, N. R. (2005). norepinephrine transporter: A candidate gene for initial ethanol sensitivity in inbred long‐sleep and short‐sleep mice. Alcoholism: Clinical and Experimental Research, 29, 1759–1768. https://doi.org/10.1097/01.alc.0000183009.57805.a6 10.1097/01.alc.0000183009.57805.a6 16269905

[brb3989-bib-0018] Heath, A. C. , Madden, P. A. , Bucholz, K. K. , Dinwiddie, S. H. , Slutske, W. S. , Bierut, L. J. , … Martin, N. G. (1999). Genetic differences in alcohol sensitivity and the inheritance of alcoholism risk. Psychological Medicine, 29, 1069–1081. https://doi.org/10.1017/S0033291799008909 10576299 10.1017/s0033291799008909

[brb3989-bib-0019] Hoffman, P. L. , Bennett, B. , Saba, L. M. , Bhave, S. V. , Carosone‐Link, P. J. , Hornbaker, C. K. , … Tabakoff, B. (2011). Using the Phenogen website for ‘in silico’ analysis of morphine‐induced analgesia: Identifying candidate genes. Addiction Biology, 16, 393–404. https://doi.org/10.1111/j.1369-1600.2010.00254.x 21054686 10.1111/j.1369-1600.2010.00254.x PMC3115429

[brb3989-bib-0020] Houston, C. M. , Lee, H. H. , Hosie, A. M. , Moss, S. J. , & Smart, T. G. (2007). Identification of the sites for CaMK‐II‐dependent phosphorylation of GABAA receptors. Journal of Biological Chemistry, 282, 17855–17865. https://doi.org/10.1074/jbc.M611533200 17442679 10.1074/jbc.M611533200

[brb3989-bib-0022] Johnson, B. A. , Ait‐Daoud, N. , Seneviratne, C. , Roache, J. D. , Javors, M. A. , Wang, X.‐Q. , … Li, M. D. (2011). Pharmacogenetic approach at the serotonin transporter gene as a method of reducing the severity of alcohol drinking. American Journal of Psychiatry, 168(3), 265–275. 21247998 10.1176/appi.ajp.2010.10050755 PMC3063997

[brb3989-bib-0024] Langmead, B. , Trapnell, C. , Pop, M. , & Salzberg, S. L. (2009). Ultrafast and memory‐efficient alignment of short DNA sequences to the human genome. Genome Biology, 10, R25 https://doi.org/10.1186/gb-2009-10-3-r25 19261174 10.1186/gb-2009-10-3-r25 PMC2690996

[brb3989-bib-0025] Lewohl, J. M. , Nunez, Y. O. , Dodd, P. R. , Tiwari, G. R. , Harris, R. A. , & Mayfield, R. D. (2011). Up‐Regulation of MicroRNAs in Brain of Human Alcoholics. Alcoholism: Clinical and Experimental Research, 35, 1928–1937. https://doi.org/10.1111/j.1530-0277.2011.01544.x 10.1111/j.1530-0277.2011.01544.x PMC3170679 21651580

[brb3989-bib-0026] Li, B. , & Dewey, C. N. (2011). RSEM: Accurate transcript quantification from RNA‐Seq data with or without a reference genome. BMC Bioinformatics, 12, 323 https://doi.org/10.1186/1471-2105-12-323 21816040 10.1186/1471-2105-12-323 PMC3163565

[brb3989-bib-0027] Lisman, J. , Yasuda, R. , & Raghavachari, S. (2012). Mechanisms of CaMKII action in long‐term potentiation. Nature Reviews Neuroscience, 13, 169–182. https://doi.org/10.1038/nrn3192 22334212 10.1038/nrn3192 PMC4050655

[brb3989-bib-0028] Little, P. J. , Kuhn, C. M. , Wilson, W. A. , & Swartzwelder, H. S. (1996). Differential effects of ethanol in adolescent and adult rats. Alcoholism: Clinical and Experimental Research, 20, 1346–1351. https://doi.org/10.1111/j.1530-0277.1996.tb01133.x 10.1111/j.1530-0277.1996.tb01133.x 8947309

[brb3989-bib-0029] Lucchesi, W. , Mizuno, K. , & Giese, K. P. (2011). Novel insights into CaMKII function and regulation during memory formation. Brain Research Bulletin, 85, 2–8. https://doi.org/10.1016/j.brainresbull.2010.10.009 21070840 10.1016/j.brainresbull.2010.10.009

[brb3989-bib-0030] Lund, S. P. , Nettleton, D. , Mccarthy, D. J. , & Smyth, G. K. (2012). Detecting differential expression in RNA‐sequence data using quasi‐likelihood with shrunken dispersion estimates. Statistical Applications in Genetics and Molecular Biology, 11, 8. 10.1515/1544-6115.1826 23104842

[brb3989-bib-0032] Mamdani, M. , Williamson, V. , Mcmichael, G. O. , Blevins, T. , Aliev, F. , Adkins, A. , … Web, B. T. (2015). Integrating mRNA and miRNA weighted gene co‐expression networks with eQTLs in the nucleus accumbens of subjects with alcohol dependence. PLoS One, 10, e0137671 https://doi.org/10.1371/journal.pone.0137671 26381263 10.1371/journal.pone.0137671 PMC4575063

[brb3989-bib-0033] Markel, P. D. , Defries, J. C. , & Johnson, T. E. (1995). Use of repeated measures in an analysis of ethanol‐induced loss of righting reflex in inbred long‐sleep and short‐sleep mice. Alcoholism: Clinical and Experimental Research, 19, 299–304. https://doi.org/10.1111/j.1530-0277.1995.tb01506.x 10.1111/j.1530-0277.1995.tb01506.x 7625561

[brb3989-bib-0034] Miranda, R. C. , Pietrzykowski, A. Z. , Tang, Y. , Sathyan, P. , Mayfield, D. , Keshavarzian, A. , … Hereld, D. (2010). MicroRNAs: Master regulators of ethanol abuse and toxicity? Alcoholism: Clinical and Experimental Research, 34, 575–587. https://doi.org/10.1111/j.1530-0277.2009.01126.x 10.1111/j.1530-0277.2009.01126.x PMC2925252 20102566

[brb3989-bib-0035] Musunuru, K. , Strong, A. , Frank‐Kamenetsky, M. , Lee, N. E. , Ahfeldt, T. , Sachs, K. V. , … Ruda, V. M. (2010). From noncoding variant to phenotype via SORT1 at the 1p13 cholesterol locus. Nature, 466, 714–719. https://doi.org/10.1038/nature09266 20686566 10.1038/nature09266 PMC3062476

[brb3989-bib-0036] Nunez, Y. O. , & Mayfield, R. D. (2012). Understanding alcoholism through microRNA signatures in brains of human alcoholics. non‐coding RNA and addiction, 17. 10.3389/fgene.2012.00043 PMC3322338 22514554

[brb3989-bib-0037] Nunez, Y. O. , Truitt, J. M. , Gorini, G. , Ponomareva, O. N. , Blednov, Y. A. , Harris, R. A. , & Mayfield, R. D. (2013). Positively correlated miRNA‐mRNA regulatory networks in mouse frontal cortex during early stages of alcohol dependence. BMC Genomics, 14, 1. 24148570 10.1186/1471-2164-14-725 PMC3924350

[brb3989-bib-0038] Olsen, R. W. , Hanchar, H. J. , Meera, P. , & Wallner, M. (2007). GABA A receptor subtypes: The “one glass of wine” receptors. Alcohol, 41, 201–209. https://doi.org/10.1016/j.alcohol.2007.04.006 17591543 10.1016/j.alcohol.2007.04.006 PMC2852584

[brb3989-bib-0039] Pasquinelli, A. E. (2012). MicroRNAs and their targets: Recognition, regulation and an emerging reciprocal relationship. Nature Reviews Genetics, 13, 271–282. https://doi.org/10.1038/nrg3162 10.1038/nrg3162 22411466

[brb3989-bib-0040] Pietrzykowski, A. Z. , Friesen, R. M. , Martin, G. E. , Puig, S. I. , Nowak, C. L. , Wynne, P. M. , … Treistman, S. N. (2008). Posttranscriptional regulation of BK channel splice variant stability by miR‐9 underlies neuroadaptation to alcohol. Neuron, 59, 274–287. https://doi.org/10.1016/j.neuron.2008.05.032 18667155 10.1016/j.neuron.2008.05.032 PMC2714263

[brb3989-bib-0041] Purves, D. , Augustine, G. J. , Fitzpatrick, D. , Hall, W. C. , Lamantia, A.‐S. , Mcnamara, J. O. , & White, L. E. (2008). Neuroscience, 4th Edition, Sunderland, MA: Sinauer Associates, Inc.

[brb3989-bib-0042] Rau, A. , Gallopin, M. , Celeux, G. , & Jaffrézic, F. (2013). Data‐based filtering for replicated high‐throughput transcriptome sequencing experiments. Bioinformatics, 29, 2146–2152. https://doi.org/10.1093/bioinformatics/btt350 23821648 10.1093/bioinformatics/btt350 PMC3740625

[brb3989-bib-0043] Rosenthal, R. (1978). Combining results of independent studies. Psychological Bulletin, 85, 185 https://doi.org/10.1037/0033-2909.85.1.185

[brb3989-bib-0044] Ru, Y. , Kechris, K. J. , Tabakoff, B. , Hoffman, P. , Radcliffe, R. A. , Bowler, R. , … Bemis, L. (2014). The multiMiR R package and database: Integration of microRNA–target interactions along with their disease and drug associations. Nucleic Acids Research, 42, e133–e133. https://doi.org/10.1093/nar/gku631 25063298 10.1093/nar/gku631 PMC4176155

[brb3989-bib-0045] Schuckit, M. A. (1980). Self‐rating of alcohol intoxication by young men with and without family histories of alcoholism. Journal of Studies on Alcohol, 41, 242–249. https://doi.org/10.15288/jsa.1980.41.242 7374142 10.15288/jsa.1980.41.242

[brb3989-bib-0046] Schuckit, M. A. (1994). Low level of response to alcohol as a predictor of future alcoholism. American Journal of Psychiatry, 151, 184–189. 8296886 10.1176/ajp.151.2.184

[brb3989-bib-0047] Shipton, O. A. , & Paulsen, O. (2014). GluN2A and GluN2B subunit‐containing NMDA receptors in hippocampal plasticity. Philosophical Transactions of the Royal Society of London. Series B, Biological Sciences, 369, 20130163. 24298164 10.1098/rstb.2013.0163 PMC3843894

[brb3989-bib-0048] Shonesy, B. C. , Jalan‐Sakrikar, N. , Cavener, V. S. , & Colbran, R. J. (2014). CaMKII: A molecular substrate for synaptic plasticity and memory. Progress in Molecular Biology and Translational Science, 122, 61–87. https://doi.org/10.1016/B978-0-12-420170-5.00003-9 24484698 10.1016/B978-0-12-420170-5.00003-9

[brb3989-bib-0049] Silveri, M. , & Spear, L. (2002). The effects of NMDA and GABAA pharmacological manipulations on ethanol sensitivity in immature and mature animals. Alcoholism: Clinical and Experimental Research, 26, 449–456. https://doi.org/10.1111/j.1530-0277.2002.tb02560.x 11981119

[brb3989-bib-0050] Skarnes, W. C. , Rosen, B. , West, A. P. , Koutsourakis, M. , Bushell, W. , Iyer, V. , … Cox, T. (2011). A conditional knockout resource for the genome‐wide study of mouse gene function. Nature, 474, 337–342. https://doi.org/10.1038/nature10163 21677750 10.1038/nature10163 PMC3572410

[brb3989-bib-0051] Smyth, G. K. (2005). Limma: Linear models for microarray data Bioinformatics and computational biology solutions using R and Bioconductor. New York: Springer.

[brb3989-bib-0052] Tabakoff, B. , & Hoffman, P. L. (2000). Animal models in alcohol research. Alcohol Research and Health, 24, 77–84. 11199281 PMC6713012

[brb3989-bib-0053] Team, R. C. (2000). R language definition. Retrieved from CRAN sites.

[brb3989-bib-0054] Vanderlinden, L. A. , Saba, L. M. , Bennett, B. , Hoffman, P. L. , & Tabakoff, B. (2015). Influence of sex on genetic regulation of “drinking in the dark” alcohol consumption. Mammalian Genome, 26, 43–56. https://doi.org/10.1007/s00335-014-9553-8 25559016 10.1007/s00335-014-9553-8 PMC4306387

[brb3989-bib-0056] Williams, R. W. , Bennett, B. , Lu, L. , Gu, J. , Defries, J. C. , Carosone–Link, P. J. , … Johnson, T. E. (2004). Genetic structure of the LXS panel of recombinant inbred mouse strains: A powerful resource for complex trait analysis. Mammalian Genome, 15, 637–647. https://doi.org/10.1007/s00335-004-2380-6 15457343 10.1007/s00335-004-2380-6

[brb3989-bib-0058] Yang, H. , Wang, J. R. , Didion, J. P. , Buus, R. J. , Bell, T. A. , Welsh, C. E. , … Pialek, J. (2011). Subspecific origin and haplotype diversity in the laboratory mouse. Nature Genetics, 43, 648–655. https://doi.org/10.1038/ng.847 21623374 10.1038/ng.847 PMC3125408

