# Supplementary material for: miRNA‐regulated transcription associated with mouse strains predisposed to hypnotic effects of ethanol
Source: Brain Behav. 2018 Apr 30;8(6):e00989. doi: 10.1002/brb3.989 (PMC5991579; doi:10.1002/brb3.989)

# miRNA-Regulated Transcription Associated with Predisposition to Sedative Effects of Ethanol in Mice

## Supplemental Figures

October 31, 2016

Figure S1: Graphical depiction of the overlap between differential expression (DE) results for the SAL and NVE datasets for features that were classified as up-regulated in ILS mice or not. On the x-axis are the p-value thresholds that were used for this classification (i.e. a miRNA is considered up-regulated in ILS if the p-value for that miRNA is less than the threshold and the DE is in the correct direction), and on the y-axis is the $-log_{10}(p)$ value from a Fisher's exact test on the $2x2$ table generated by classifying miRNAs as up-regulated in ILS or not in the two datasets. The horizontal black line represents a p-value of 0.05

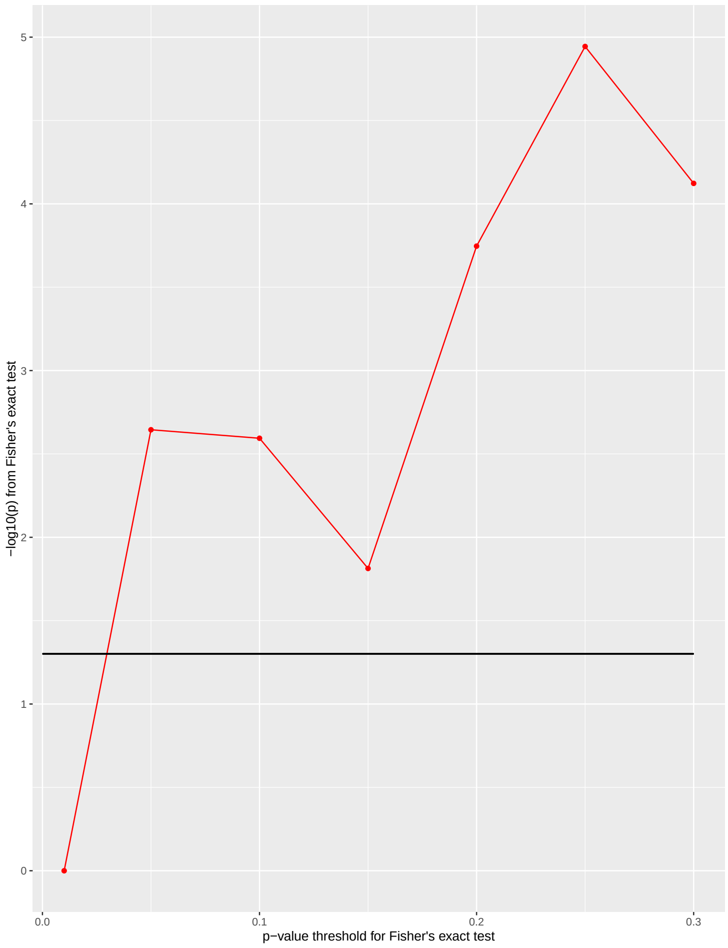

Figure S2: Graphical depiction of the overlap between differential expression (DE) results for the SAL and NVE datasets for features that were classified as up-regulated in ISS mice or not. On the x-axis are the p-value thresholds that were used for this classification (i.e. a miRNA is considered up-regulated in ISS if the p-value for that miRNA is less than the threshold and the DE is in the correct direction), and on the y-axis is the $-log_{10}(p)$ value from a Fisher's exact test on the $2x2$ table generated by classifying miRNAs as up-regulated in ISS or not in the two datasets. The horizontal black line represents a p-value of 0.05

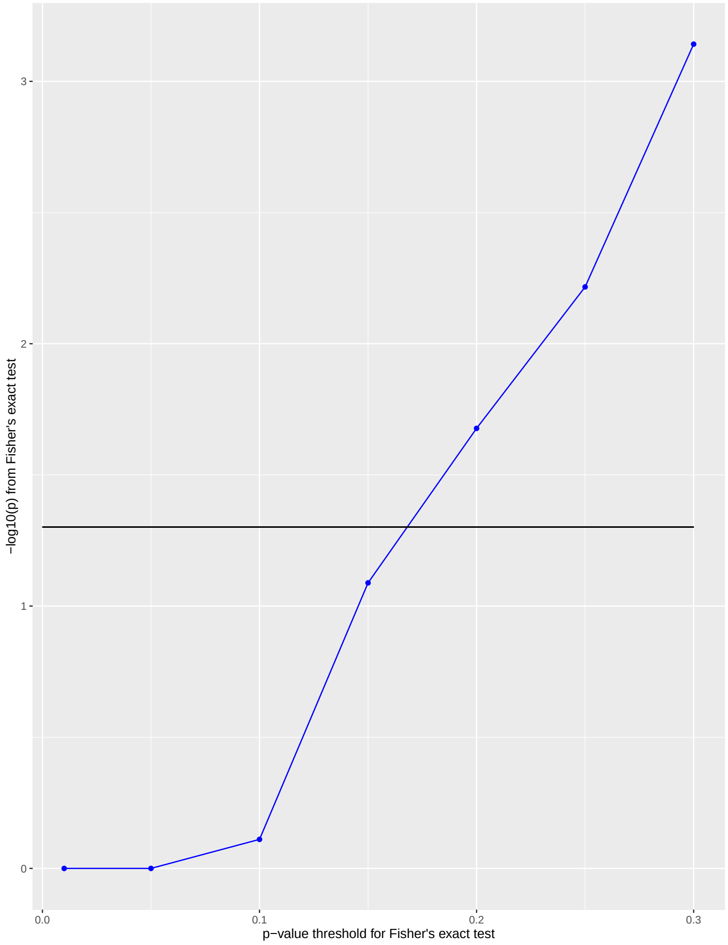

Supplement: Supplementary file 1 [file BRB3-8-e00989-s001.pdf]
