# Supplementary material for: miRNA‐regulated transcription associated with mouse strains predisposed to hypnotic effects of ethanol
Source: Brain Behav. 2018 Apr 30;8(6):e00989. doi: 10.1002/brb3.989 (PMC5991579; doi:10.1002/brb3.989)
Supplement: Supplementary file 10 [file BRB3-8-e00989-s010.docx]

**Supplementary Methods and Results**

Details on miRNA Quantitation

For both small RNA sequencing data sets (Figure 1A-B), the Fastx toolkit version 0.0.13 was used to clip adapters and filter reads for quality (>95% Q20 bases) (Gordon and Hannon, 2010).Since only size selection was used in the library preparation, some reads aligned to non-miRNA features. To compare the proportion of reads aligned to miRNAs to the proportion of reads aligned elsewhere in the genome, samples were aligned using Bowtie (version 1.1.1; (Langmead et al., 2009) to a transcript database consisting of all mouse transcripts in RefSeq starting with “NM” and “NR” identifiers (Release 67; (Pruitt et al., 2007). Because miRNAs are the main focus of this manuscript, quantification for statistical analyses was performed with RSEM (version 1.2.19; (Li and Dewey, 2011) to estimate counts for a miRNA transcriptome consisting of the mouse miRBase database (version 21) for mature miRNA transcripts (Li and Dewey, 2011, Griffiths-Jones et al., 2006).

Details on mRNA Quantitation

For measures of brain mRNA expression levels, the public data set on 60 LXS strains and the two parental strains (n=4 to 6 mice per strain) was downloaded in the form of Affymetrix Mouse Exon 1.0 ST Array (Affymetrix, Santa Clara, CA) CEL files from the PhenoGen website (<http://phenogen.ucdenver.edu>;(Vanderlinden et al., 2015, Hoffman et al., 2011). The probe mask described previously in (Vanderlinden et al., 2015) was used to eliminate low integrity probes, i.e., probes that did not align uniquely to the mm10 version of the mouse genome or aligned to a region of the genome that harbored a sequence polymorphism between either parental strain and the C57BL/6J reference strain. The remaining probe sets were compared to the Ensembl GRCm38/mm10 version of the transcriptome in mouse. Probe sets targeting the same Ensembl gene were aggregated into a single expression estimate for each sample using the rma-sketch pipeline for normalization and aggregation in Affymetrix Power Tools (Irizarry et al., 2003, Shakya et al., 2010). Normalized expression estimates were adjusted for batch effects using ComBat (Johnson et al., 2007) and all results are reported at the gene level.

Information on LXS Marker Set

The original LXS genetic marker set consisting of 303,988 SNPs with unique dbSNP identifiers was generated by The Jackson Laboratory using the Affymetrix Mouse Diversity Genotyping Array (Yang et al., 2011). The quality control process and the reduction of high quality informative SNPs to unique strain distribution patterns (SDPs) are described in detail in (Vanderlinden et al., 2015) including the requirement of homozygous genotype calls in the two parental strains and in 95% of the RI strains. For the QTL analysis of sleep time, the original SNP set was reduced to 1,435 unique strain distribution patterns (SDPs) for the entire LXS panel. For the eQTL analysis, we used a set of 1,414 SDPs (based only on the 60 LXS strains used for the exon array analysis) described in (Vanderlinden et al., 2015).

Differential expression of miRNAs

Filtered miRNAs were analyzed with the QuasiSeq package (V1.0-8) in R (Lund et al., 2012) using a negative binomial generalized linear model (GLM) framework where the 75th percentile of counts for a given sample was used as an offset to account for differences between library sizes across samples.

Power Simulation for Meta-Analysis Method

A simulation study was conducted to compare the statistical power for detecting differential expression between the meta-analysis method used for combining the miRNA results between SAL and NVE mice and what could have been achieved if both data sets could have been analyzed in a single model. To do this, data sets were simulated using the same negative binomial generalized linear model framework that is assumed for the analysis of the RNA-Seq data using QuasiSeq. Here, the observed counts for gene *i* in sample *k* are denoted by Y_ij_ and are assumed to follow a negative binomial distribution with mean $\mu_{ij}$ and dispersion $\rho_{i}$. The mean and covariates are linked via the relationship

$\log\left( \mu_{ij} \right)= \beta_{0i}+ \beta_{1i}\cdot x_{j}$

where x_j_ is equal to 1 if sample *j* is from an ISS mouse and 0 if it is from an ILS mouse. That is, $\beta_{1i}$ is the differential expression effect (on the log scale) for gene *i*. For each effect size examined, 20 simulated data sets were generated with 500 genes (all differentially expressed) and 12 samples (6 ISS and 6 ILS) each. The intercept values $\beta_{0i}$ were simulated from a uniform (5, 12) distribution while dispersion values were simulated from a uniform (0.25, 5) distribution. Given these values, observed counts were then simulated from a negative binomial ($\mu_{ij}$, $\rho_{i})$ distribution for each gene in each sample. To estimate the power from the meta-analysis technique, the 12 samples were separated into 2 smaller data sets (i.e. a SAL and a NVE set), each with 3 ISS samples and 3 ILS samples. Differential expression was determined using the same QuasiSeq analysis pipeline that was used on the real data, and the meta-analysis was applied to the resulting p-values to determine a meta-analysis p-value. Power was estimated as the proportion of genes with a meta-analysis p-value < 0.05 for each data set, and a mean was taken across all 20 data sets for a given effect size. Power for the single model was done in a similar way, but this time differential expression was determined using QuasiSeq with all 12 samples at once. The results for a range of effect sizes (0.25 – 3) are in Supplemental Table 8. The meta-analysis achieved between 93%-100% of the power that could have been obtained using a single model (had that been possible), and for effect sizes that gave 80%-90% power, the meta-analysis and the single model were virtually identical.

Comparison of Saline Treated and Naïve Differentially Expressed miRNA

Although the magnitude of the overlap between the the SAL and NVE differentially expressed miRNA was not large due to our strict criteria (p-value < 0.05 for both sets), there was overall concordance between the two studies when we examined miRNAs that showed evidence of differential expression using more relaxed thresholds. For example, by classifying miRNA as either significantly up-regulated in ILS (p-value < 0.20 and DE in the correct direction) or not significantly up-regulated in ILS, there was a significant association between the two studies in their classification of the miRNAs (Fisher’s exact test p-value = 1.8e-4). Performing this same process but instead classifying miRNAs as either up-regulated in ISS or not gave similar results (Fisher’s exact test p-value = 0.02). Supplemental Figures 1 and 2 show the trend as the p-value threshold for defining a DE miRNA ranges between 0.01-0.30.

Additionally, we compared the overall miRNA expression profiles (post counts per million filtering) for the 12 mice included in this study by computing all pairwise Spearman correlations between the samples. The minimum correlation between two samples was 0.91 while the bulk of the correlations were around 0.95-0.98. That is, the two most uncorrelated samples in the data still had an overall correlation of 0.91 in terms of miRNA expression. This seems to suggest that the overall expression profiles are in fact quite similar across both the NVE and SAL mice, and also seems to replicate the above analysis investigating overlap in differential expression testing results.

Details on Integrative Analysis of miRNAs and mRNAs

Prior to combing the p-values from the three different analyses (miRNA differential expression, mRNA differential expression, mRNA-LORR correlation), extreme values of the Z-statistics converted from the p-values obtained in the mRNA differential expression analyses and the mRNA-LORR correlation analyses were Winsorized (Dixon and Tukey, 1968) at roughly the magnitude of the largest Z-statistic from the miRNA differential expression analyses in an effort to temper the influence of the two mRNA-based analyses on the integrative meta-analysis. This was done to avoid candidate miRNA-mRNA pairs that showed no evidence of differential expression in the miRNA but extreme differences in mRNA expression. Additionally, the directions of the individual effects were accounted for to focus on pairs that showed evidence of a direct relationship (up regulation in the miRNAs and down regulation of the target mRNAs and vice versa). Finally, the resulting meta-analysis p-value was adjusted for multiple comparisons and significant pairs were identified using a FDR threshold of 0.10.

Details on Characterization of miRNA-mRNA pairs: Pathway Enrichment

We performed two separate pathway analyses. First, examining mRNAs that are targeted by our top miRNAs but using other mRNA target resources, not only the mRNAs in our top pairs. Then, we examined the mRNAs in our top pairs (i.e., LORR associated mRNAs that show evidence of miRNA targeting) and examined enriched pathways that may be different from enrichment in all mRNAs associated with LORR (but may not be targeted by miRNAs). The two pathway enrichment strategies were necessary because not all mRNAs are represented on the exon arrays, either because they were not on the array or removed through filtering. Therefore, we did not want to limit the pathway analysis to only the top mRNAs in our pairs. The two sets of pathway results provided different but complementary results related to neuron synapse and function.

*mRNA Pathway Analysis:* All of the unique mRNAs in the significant miRNA-mRNA pairs from the integrative meta-analysis were tested for statistical enrichment versus the background set of all mRNAs associated with LORR (i.e. mRNAs with an FDR < 0.05 in the DE analysis of mRNA features in 2.3.2) to explore if there was functional enrichment due to miRNA regulation. This was performed using the Panther web tool V10 (Mi et al., 2016) for each of Panther pathways (Nikolsky and Bryant, 2009), GO molecular functions, and GO biological processes (Ashburner et al., 2000). To determine significance under each paradigm, the standard output was filtered to include only those gene sets (i.e., pathways, functions or processes) that had at least 2 genes from the list of miRNA targeted differentially expressed mRNAs. From this, a p-value for overrepresentation was obtained for each remaining feature, and these were then adjusted for multiple comparisons (FDR < 0.10).

*miRNA Pathway Analysis:* A similar process was performed for the unique set of miRNAs in the significant pairs using the miRpath tool v3.0 (Vlachos et al., 2015). Here the union of the predicted targets from microT-CDS v5.0 (Paraskevopoulou et al., 2013) and the validated targets from TarBase v7.0 (Vlachos et al., 2014)for each miRNA were analyzed for overrepresentation of KEGG pathways (FDR < 0.05). This was done for all miRNAs involved in the significant miRNA-mRNA pairs simultaneously, and then for a selected subset of highly connected (i.e. at least 5 mRNA targets in the significant miRNA-mRNA pairs) miRNAs individually.

Details on Characterization of miRNA-mRNA pairs: eQTL

Location of the miRNAs in the genome was compared to both the associated mRNA’s expression (e)QTL (if a significant one was found) and the LORR QTLs.

The eQTL were calculated using weighted marker regression (Carlborg et al., 2005) on strain means for individual Ensembl genes. Genome-wide p-values were calculated empirically through permutations limited to unique SDP (Churchill and Doerge, 1994). Confidence intervals for QTL location were calculated using the bootstrap methods outlined in (Visscher et al., 1996). All eQTL calculations were conducted in QTLReaper (http://qtlreaper.sourceforge.net/).

The LORR QTLs were calculated using marker regression on strain means with genome-wide p-values calculated empirically. Confidence intervals for LORR QTL location are represented by a 90% Bayesian credible interval (Sen and Churchill, 2001). All calculations for LORR QTL were executed in the R/qtl package of R statistical software (Broman et al., 2003). Additionally, the location within the genome of each miRNA was compared to several LORR QTLs discovered in previous studies (Markel et al., 1997, Bennett et al., 2015).

Details on Characterization of miRNA-mRNA pairs: Summaries

Other summaries that were tabulated for the significant pairs included: the number of mRNAs targeted by each miRNA, the number of miRNAs that targeted each mRNA, the number of functional gene sets (from the Panther web tool described above) that each miRNA targeted (defined by targeting at least one mRNA in that set), and for each functional gene set that a miRNA targeted, how many of the genes in that gene set were targeted, and which KEGG pathways were overrepresented among all target mRNAs for each miRNA based on the mirPath analysis.

Validaton of Selected Predicted Target

The first construct contained the predicted binding site and the second construct contained site directed mutations in the predicted binding site as previously described (Pillai et al., 2010). Plasmids were transfected into HEK293T cells using Lipofectamine 2000 (ThermoFisher Scientific) and treated with a control mimic (Allstars negative control, Qiagen) or a specific mimic of miR-106b-5p (Qiagen). Following 12 hours of incubation, luciferase activity was quantitated on a luminometer and compared to the internal control in the dual luciferase vector for transfection efficiency.

For-CAM106b

5’- tcgaAGGAGGAGGAGGGAGAAAGCACTTTGAAATTTATTAATAGCTTGCTACCTGCG

and Rev-CAM106b

5’- ggccCGC AGG TAG CAA GCT ATT AAT AAA TTT CAA AGT GCT TTC TCC CTC CTC CTC CT

and for the mutant construct For-CAM106b

mut 5’- tcgaAGGAGGAGGAGGGAGAAAGCAtTcTGAAATTTATTAATAGCTTGCTACCTGCG

and Rev-CAM106b

mut 5’- ggccCGC AGG TAG CAA GCT ATT AAT AAA TTT CAg AaT GCT TTC TCC CTC CTC CTC CT

Exploring Concordance

Our miRNA-specific analysis did not result in a large number of miRNAs that showed significant differential expression in both the saline treated and naïve data sets. This could in part be due to the stringency of our methods and lack of power in each independent analysis (only 3 samples per strain in each dataset), differences in sequencing depth (higher in the naïve set), and somewhat different dissection methods (cerebellum and olfactory bulbs were included in the naive set but not the saline treated set). Despite the small number of overlapping miRNA, there was overall concordance in the results between the two studies if we relaxed our criteria. However, the use of the meta-analysis to combine the individual results gave a larger number of features to interrogate than either of the individual analyses. Moreover, a simulation study (details previously described) showed that the meta-analysis technique gave statistical power close to what could have been achieved for a similar analysis using n=6 in the ILS and ISS strains. Indeed, for log-effect sizes between 0.25-3, the meta-analysis gave power that was between 93%-100% of what would have been achieved using a single model with n=6 in each strain.

Additional Considerations

While a lack of additional samples on which to perform qRT-PCR validation for expression levels is a limitation and should be the subject of future research, the fact that the miRNA differential expression analysis was only one of four pieces that went into the integrative meta-analysis (mRNA DE, mRNA-LORR correlation, miRNA-mRNA target prediction) provides additional confidence in the overall results. For the mRNA specific analyses, not all genes were represented on the exon arrays, either because they were removed through filtering or they were never there to begin with. Additionally, the LORR correlation results showed weaker signal than the differential expression results between the ILS and ISS. This is not unexpected since the ILS and ISS are at the extremes of the LORR distribution so they should show the most dramatic changes. The ILS and ISS strains also differ on many other traits in addition to ethanol sensitivity, including alcohol intake and withdrawal symptoms (Church et al., 1979, Gilliam and Collins, 1986). Moreover, the injection procedure performed on the saline treated mice may itself alter gene or miRNA expression. However, we do not expect the expression of the genes/miRNAs associated with LORR to be affected since the phenotype itself does not appear to be sensitive to the injection.

As a first approach, this study only focused on male mice because the analysis of data from female mice poses challenges since gene expression is known to fluctuate during the ovarian cycle (Yang et al., 2006, Maguire et al., 2005). Future studies in females would require the synchronization of ovarian cycles among female mice. Based on our experience, the ILS mice have especially inconsistent ovarian cycles, which will need to be considered. Regarding phenotypic variation between male and females, while there are differences in LORR values observed between male and female mice from the same LXS strain, the correlation between males and females is fairly strong (Spearman’s rank-correlation = 0.84; (Bennett et al., 2006). This high correlation suggests that although our study was specific to male mice, the results provide candidate miRNA-mRNA for future evaluation in females. Additionally, previous studies have found that gene expression does not significantly differ between the left and right hemispheres of the brain in rodents, so we do not expect any biases to be introduced by using the right hemisphere for miRNA expression and the left for mRNA expression in the naïve mice (Brown et al., 2002).

Although there is evidence of miRNA and gene expression differences among brain regions (Bak et al., 2008, Hovatta et al., 2007), we have chosen to emphasize the whole brain at this stage for several reasons. First, for complex diseases such as alcoholism, the brain region most responsible for the trait may not be known or there may be multiple regions involved with interactions among the regions that are not well defined (Koob and Volkow, 2010) . Therefore, whole brain profiling provides a more general resource when the relevant brain regions are unknown or there are not enough resources to profile multiple brain regions. Second, brain regions, such as the hippocampus, can still be quite heterogeneous. This poses practical challenges for the scope of the study regarding the appropriate level of dissection. Third, work in our group comparing whole brain gene expression data with brain regional data indicates that for gene co-expression, results that are consistent across brain regions can still be identified using whole brain analysis (Vanderlinden et al., 2013). Finally, our method is based on existing gene expression data on the entire LXS panel, which at this time only includes whole brain or hippocampus for naïve mice. Although some evidence supports the role of the hippocampus in ethanol tolerance, other regions have been implicated for tolerance such as the striatum, and the role of the hippocampus for the other traits is still unclear. In summary, although whole brain analysis may result in false negatives, the large number of true positives that can be found with whole brain data should outweigh this drawback.

ASHBURNER, M., BALL, C. A., BLAKE, J. A., BOTSTEIN, D., BUTLER, H., CHERRY, J. M., DAVIS, A. P., DOLINSKI, K., DWIGHT, S. S. & EPPIG, J. T. 2000. Gene Ontology: tool for the unification of biology. *Nature genetics,* 25**,** 25-29.

BAK, M., SILAHTAROGLU, A., MØLLER, M., CHRISTENSEN, M., RATH, M. F., SKRYABIN, B., TOMMERUP, N. & KAUPPINEN, S. 2008. MicroRNA expression in the adult mouse central nervous system. *Rna,* 14**,** 432-444.

BENNETT, B., CAROSONE-LINK, P., ZAHNISER, N. R. & JOHNSON, T. E. 2006. Confirmation and fine mapping of ethanol sensitivity quantitative trait loci, and candidate gene testing in the LXS recombinant inbred mice. *Journal of Pharmacology and Experimental Therapeutics,* 319**,** 299-307.

BENNETT, B., LARSON, C., RICHMOND, P. A., ODELL, A. T., SABA, L. M., TABAKOFF, B., DOWELL, R. & RADCLIFFE, R. A. 2015. Quantitative Trait Locus Mapping of Acute Functional Tolerance in the LXS Recombinant Inbred Strains. *Alcoholism: Clinical and Experimental Research,* 39**,** 611-620.

BROMAN, K. W., WU, H., SEN, Ś. & CHURCHILL, G. A. 2003. R/qtl: QTL mapping in experimental crosses. *Bioinformatics,* 19**,** 889-890.

BROWN, V. M., OSSADTCHI, A., KHAN, A. H., YEE, S., LACAN, G., MELEGA, W. P., CHERRY, S. R., LEAHY, R. M. & SMITH, D. J. 2002. Multiplex three-dimensional brain gene expression mapping in a mouse model of Parkinson's disease. *Genome research,* 12**,** 868-884.

CARLBORG, Ö., DE KONING, D., MANLY, K. F., CHESLER, E., WILLIAMS, R. W. & HALEY, C. S. 2005. Methodological aspects of the genetic dissection of gene expression. *Bioinformatics,* 21**,** 2383-2393.

CHURCH, A. C., FULLER, J. L. & DANN, L. 1979. Alcohol intake in selected lines of mice: Importance of sex and genotype. *Journal of comparative and physiological psychology,* 93**,** 242.

CHURCHILL, G. A. & DOERGE, R. W. 1994. Empirical threshold values for quantitative trait mapping. *Genetics,* 138**,** 963-971.

DIXON, W. J. & TUKEY, J. W. 1968. Approximate behavior of the distribution of Winsorized t (Trimming/Winsorization 2). *Technometrics,* 10**,** 83-98.

GILLIAM, D. M. & COLLINS, A. C. 1986. Quantification of Physiological and Behavioral Measures of Alcohol Withdrawal in Long‐Sleep and Short‐Sleep Mice. *Alcoholism: Clinical and Experimental Research,* 10**,** 672-678.

GORDON, A. & HANNON, G. 2010. Fastx-toolkit. *FASTQ/A short-reads preprocessing tools (unpublished)* <http://hannonlab>*. cshl. edu/fastx_toolkit*.

GRIFFITHS-JONES, S., GROCOCK, R. J., VAN DONGEN, S., BATEMAN, A. & ENRIGHT, A. J. 2006. miRBase: microRNA sequences, targets and gene nomenclature. *Nucleic acids research,* 34**,** D140-D144.

HOFFMAN, P. L., BENNETT, B., SABA, L. M., BHAVE, S. V., CAROSONE‐LINK, P. J., HORNBAKER, C. K., KECHRIS, K. J., WILLIAMS, R. W. & TABAKOFF, B. 2011. Using the Phenogen website for ‘in silico’analysis of morphine‐induced analgesia: identifying candidate genes. *Addiction biology,* 16**,** 393-404.

HOVATTA, I., ZAPALA, M. A., BROIDE, R. S., SCHADT, E. E., LIBIGER, O., SCHORK, N. J., LOCKHART, D. J. & BARLOW, C. 2007. DNA variation and brain region-specific expression profiles exhibit different relationships between inbred mouse strains: implications for eQTL mapping studies. *Genome biology,* 8**,** 1.

IRIZARRY, R. A., BOLSTAD, B. M., COLLIN, F., COPE, L. M., HOBBS, B. & SPEED, T. P. 2003. Summaries of Affymetrix GeneChip probe level data. *Nucleic acids research,* 31**,** e15-e15.

JOHNSON, W. E., LI, C. & RABINOVIC, A. 2007. Adjusting batch effects in microarray expression data using empirical Bayes methods. *Biostatistics,* 8**,** 118-127.

KOOB, G. F. & VOLKOW, N. D. 2010. Neurocircuitry of addiction. *Neuropsychopharmacology,* 35**,** 217-238.

LANGMEAD, B., TRAPNELL, C., POP, M. & SALZBERG, S. L. 2009. Ultrafast and memory-efficient alignment of short DNA sequences to the human genome. *Genome biol,* 10**,** R25.

LI, B. & DEWEY, C. N. 2011. RSEM: accurate transcript quantification from RNA-Seq data with or without a reference genome. *BMC bioinformatics,* 12**,** 323.

LUND, S. P., NETTLETON, D., MCCARTHY, D. J. & SMYTH, G. K. 2012. Detecting differential expression in RNA-sequence data using quasi-likelihood with shrunken dispersion estimates. *Statistical applications in genetics and molecular biology,* 11**,** 8.

MAGUIRE, J. L., STELL, B. M., RAFIZADEH, M. & MODY, I. 2005. Ovarian cycle–linked changes in GABAA receptors mediating tonic inhibition alter seizure susceptibility and anxiety. *Nature neuroscience,* 8**,** 797-804.

MARKEL, P. D., BENNETT, B., BEESON, M., GORDON, L. & JOHNSON, T. E. 1997. Confirmation of quantitative trait loci for ethanol sensitivity in long-sleep and short-sleep mice. *Genome research,* 7**,** 92-99.

MI, H., POUDEL, S., MURUGANUJAN, A., CASAGRANDE, J. T. & THOMAS, P. D. 2016. PANTHER version 10: expanded protein families and functions, and analysis tools. *Nucleic acids research,* 44**,** D336-D342.

NIKOLSKY, Y. & BRYANT, J. 2009. Protein networks and pathway analysis.

PARASKEVOPOULOU, M. D., GEORGAKILAS, G., KOSTOULAS, N., VLACHOS, I. S., VERGOULIS, T., RECZKO, M., FILIPPIDIS, C., DALAMAGAS, T. & HATZIGEORGIOU, A. G. 2013. DIANA-microT web server v5. 0: service integration into miRNA functional analysis workflows. *Nucleic acids research***,** gkt393.

PRUITT, K. D., TATUSOVA, T. & MAGLOTT, D. R. 2007. NCBI reference sequences (RefSeq): a curated non-redundant sequence database of genomes, transcripts and proteins. *Nucleic acids research,* 35**,** D61-D65.

SEN, Ś. & CHURCHILL, G. A. 2001. A statistical framework for quantitative trait mapping. *Genetics,* 159**,** 371-387.

SHAKYA, K., RUSKIN, H., KERR, G., CRANE, M. & BECKER, J. 2010. Comparison of microarray preprocessing methods. *Advances in Computational Biology.* Springer.

VANDERLINDEN, L. A., SABA, L. M., BENNETT, B., HOFFMAN, P. L. & TABAKOFF, B. 2015. Influence of sex on genetic regulation of “drinking in the dark” alcohol consumption. *Mammalian genome,* 26**,** 43-56.

VANDERLINDEN, L. A., SABA, L. M., KECHRIS, K., MILES, M. F., HOFFMAN, P. L. & TABAKOFF, B. 2013. Whole brain and brain regional coexpression network interactions associated with predisposition to alcohol consumption. *PloS one,* 8**,** e68878.

VISSCHER, P. M., THOMPSON, R. & HALEY, C. S. 1996. Confidence intervals in QTL mapping by bootstrapping. *Genetics,* 143**,** 1013-1020.

VLACHOS, I. S., PARASKEVOPOULOU, M. D., KARAGKOUNI, D., GEORGAKILAS, G., VERGOULIS, T., KANELLOS, I., ANASTASOPOULOS, I.-L., MANIOU, S., KARATHANOU, K. & KALFAKAKOU, D. 2014. DIANA-TarBase v7. 0: indexing more than half a million experimentally supported miRNA: mRNA interactions. *Nucleic acids research***,** gku1215.

VLACHOS, I. S., ZAGGANAS, K., PARASKEVOPOULOU, M. D., GEORGAKILAS, G., KARAGKOUNI, D., VERGOULIS, T., DALAMAGAS, T. & HATZIGEORGIOU, A. G. 2015. DIANA-miRPath v3. 0: deciphering microRNA function with experimental support. *Nucleic acids research***,** gkv403.

YANG, H., WANG, J. R., DIDION, J. P., BUUS, R. J., BELL, T. A., WELSH, C. E., BONHOMME, F., YU, A. H.-T., NACHMAN, M. W. & PIALEK, J. 2011. Subspecific origin and haplotype diversity in the laboratory mouse. *Nature genetics,* 43**,** 648-655.

YANG, X., SCHADT, E. E., WANG, S., WANG, H., ARNOLD, A. P., INGRAM-DRAKE, L., DRAKE, T. A. & LUSIS, A. J. 2006. Tissue-specific expression and regulation of sexually dimorphic genes in mice. *Genome research,* 16**,** 995-1004.
